# Effective phosphorus removal using transformed water hyacinth: Performance evaluation in fixed-bed columns and practical applications

**DOI:** 10.1371/journal.pone.0312432

**Published:** 2024-11-21

**Authors:** Anyi Ramirez-Muñoz, Elizabeth Flórez, Raúl Ocampo-Perez, Nancy Acelas

**Affiliations:** 1 Grupo de Investigación Materiales con Impacto (Mat&mpac), Facultad de Ciencias Básicas, Universidad de Medellín, Medellín, Colombia; 2 Laboratorio Nacional de Proyección Térmica (CENAPROT), Centro de Investigación y de Estudios Avanzados Del IPN, Querétaro, México; 3 Centro de Investigación y de Estudios de Posgrado, Facultad de Ciencias Químicas, Universidad Autónoma de San Luis Potosí, San Luis Potosí, México; King Saud University, SAUDI ARABIA

## Abstract

This study introduces calcined water hyacinth (CWH), processed at 650°C, as a novel and environmentally friendly adsorbent for phosphorus (P) removal from wastewater. Building on previous findings that identified CWH as a rich source of metal oxides and hydroxides (e.g., Ca(OH)₂, Al₂O₃, MgO, Fe₃O₄), this research explores its application in fixed-bed column systems for continuous adsorption processes. The study demonstrates that CWH effectively removes phosphorus through apatite formation, showcasing its potential for real-world water treatment. The phosphorus adsorption capacity increased from 23.64 to 26.55 mg/g when the flow rate was reduced from 1.5 to 0.5 mL/min. Breakthrough curves fitted to the Thomas, Adams-Bohart, and Yoon-Nelson models provided critical insights into column performance, while the Bed Depth Service Time (BDST) model confirmed the feasibility of employing CWH in continuous-flow systems. The practical tests on synthetic municipal wastewater, which revealed a maximum adsorption capacity of 5.20 mg/g, further demonstrated CWH’s effectiveness for treating wastewater with low phosphorus concentrations, providing reassurance about its real-world applicability. Furthermore, the study found that increasing the adsorbent height improved column performance by extending breakthrough and exhaustion times, whereas higher flow rates led to faster saturation and reduced capacity. The exhausted CWH material can be repurposed as a soil amendment or fertilizer feedstock, supporting nutrient recycling.

## Introduction

Phosphorus (P) is a vital macronutrient essential for life and important across various industries. However, the excessive release of P stemming from industrial, domestic, and agricultural activities, as well as its leaching from soil post-fertilizer application, poses significant environmental challenges, notably eutrophication. This phenomenon triggers a rapid proliferation of algae and other planktonic organisms, leading to reduced dissolved oxygen levels and heightened mortality rates among aquatic life forms [1–6]. Consequently, this deterioration in water quality perpetuates resource degradation, profoundly impacting the ecosystem’s health. Harmful algal blooms stemming from eutrophication can contaminate drinking water sources, posing health hazards to both humans and animals. Certain algal toxins are associated with gastrointestinal illness, liver damage, neurological disorders, and skin irritation [7]. Hence, the imperative lies in removing and recovering P from wastewater, which is crucial to mitigating eutrophication and addressing phosphate rock depletion. While no universal consensus exists on acceptable P concentration, a total P concentration exceeding 0.05 mg/L is widely deemed excessive for aquatic ecosystems [8]. Consequently, numerous studies have explored various techniques for P removal and recovery from water. Among these, adsorption [9–12] emerges as a desirable methodology owing to its cost-effectiveness, seamless integration into existing systems, high efficiency, and environmentally friendly nature. The high versatility of this technology resides in the wide variety of adsorbent materials, which can be derived from biomass residues. These include biocomposites like eggshells combined with banana peels [13], orange peels [14], potato peels [15], or fiber palm [16, 17], along with readily available resources such as water hyacinth [18, 19], among other innovative sources [10–12, 18, 20].

Water hyacinth (WH) is one of the most troublesome aquatic weeds globally, renowned for its rapid growth rate [21]. Capable of forming dense volumes exceeding 60 kg/m^2^, WH plants can severely obstruct watercourses [22], disrupting aquatic ecosystems by reducing sunlight penetration and diminishing optimal conditions for aquatic life, including a decline in dissolved oxygen levels [23]. Furthermore, the presence of WH can inflict negative economic repercussions on industries reliant on water bodies, such as tourism [24]. Consequently, WH removal and effective reuse pose significant challenges for these ecosystems. Thus, the task of removing and efficiently reusing water hyacinth is not without its challenges, posing significant hurdles for these ecosystems. However, the potential for repurposing the harvested water hyacinth is vast, offering a multitude of opportunities. These include biofuel production, composting, animal feed, handicrafts, bioremediation, phytoremediation, and biogas production [25]. These avenues not only contribute to economic growth but also offer significant environmental benefits. Moreover, the ongoing research on phosphorus adsorption using biochar derived from water hyacinth pyrolysis, with modifications involving various metal oxides such as Fe, Ca, Al, and Mg, shows potential for more sustainable solutions [18]. The metal phases present in these biochars are mainly responsible for phosphorus removal, facilitating ligand exchange through the formation of Metal–O–P bonds, while precipitation of phosphorus species has been identified as a predominant adsorption mechanism. Interestingly, among the various metals present in these biomasses, calcium has demonstrated remarkable efficacy in recovering phosphorus from aqueous solutions due to its favorable formation of apatite (Ca_5_(PO_4_)_3_OH) [13, 14, 18]. Apatite, a calcium phosphate biomaterial, represents a valuable pathway for phosphorus recovery and has been employed as a soil amendment. In our previous study [23], we determined that WH ashes contain metal oxides and hydroxides, such as Ca(OH)_2_, Al_2_O_3_, MgO, and Fe_3_O_4_. These play a crucial role as active phases for phosphorus adsorption and serve as active phases for phosphorus adsorption, as apatite [18]. However, previous applications of WH for P removal and recovery have primarily focused on batch experiments [18, 26, 27]. While these studies offer valuable insights into adsorption characteristics such as mechanism, maximum adsorption capacities, optimal conditions of pH and temperature, and behavior of the system as a function of time (adsorption kinetics), they are limited in their ability to simulate the dynamic performance of adsorbers. Hence, fixed-bed column studies are crucial for scaling from laboratory to industrial applications [3, 28]. Fixed-bed column experiments are crucial for scaling up from laboratory to industrial applications, as they enable: a. Design and operational development of large-scale adsorption processes. b. Insights into breakthrough curves, determining the functional lifespan of the adsorbent bed. c. Handling large volumes of wastewater under continuous flow. d. Potential to achieve high treatment efficiencies [29, 30].

Therefore, this work’s novelty lies in conducting fixed-bed column studies, which provide valuable data for translating laboratory findings to practical industrial-scale adsorption systems, bridging the gap between batch and continuous flow conditions. Additionally, this study utilizes water hyacinth as an adsorbent, creating a valuable product from waste biomass and effectively addressing disposal issues.

While previous studies have explored the adsorption characteristics of Water Hyacinth (WH) through batch experiments, more detailed investigations are needed into its continuous adsorption performance under dynamic flow conditions. This gap underscores the necessity for further research into its potential scale industrial applications. Accordingly, this study aims to evaluate the performance of Calcined Water Hyacinth (CWH) in continuous adsorption experiments specifically for phosphorus (P) removal. The primary objective is to identify the optimal operating conditions that effectively reduce the initial phosphorus concentration. Additionally, the study investigates breakthrough curves to understand how bed height, initial phosphorus concentration, and flow rate influence adsorption in fixed-bed columns. To analyze the column adsorption data and predict breakthrough curves, a range of widely used models—including the Thomas, Adams-Bohart, Yoon-Nelson, and Bed Depth Service Time (BDST) models—were employed. Furthermore, the study explores the practical feasibility of using CWH for phosphorus removal from synthetic municipal wastewater, providing valuable insights into its potential real-world applications and contributing to advancements in wastewater treatment technologies. This study promises a significant impact in the field, offering hope for improved wastewater treatment methods.

## Materials and methods

### Adsorbent material

The aquatic weed Water Hyacinth was harvested from the Cauca River at the Ituango Hydroelectric Plant (Hidroituango), situated between the municipalities of Ituango and Briceño, in the department of Antioquia, Colombia. A composite of all parts of the harvested Water Hyacinth, including roots, stems, and leaves, was subjected to a calcination process to yield the Calcined Water Hyacinth (CWH) adsorbent material. In our previous study [18] the sample, referred to as WH, was washed with distilled water to remove impurities, dried in an oven at 105°C for 1440 min, ground, and finally passed through a sieve with a size < 0.850 mm. The water hyacinth (WH) sample was calcined in a horizontal tube furnace at 350°C, 450°C, 550°C, 600°C, 650°C, and 700°C, with a heating rate of 10°C/min, and held at each temperature for 120 minutes in an air atmosphere (100 mL/min). The resulting materials were ground and sieved to a particle size of 60–100 mesh. All chemical reagents used were of analytical grade (see supplementary material). In our previous research [18], we explored phosphorus adsorption using materials calcined at these various temperatures. Our findings indicated that the material calcined at 650°C (yield = 16.7%) demonstrated the highest adsorption efficiency, primarily due to the formation of Ca(OH)₂, Al₂O₃, and MgO phases, which are known to enhance phosphorus removal. Thus, the material calcined at 650°C was selected as the focus of the current study. The material before (CWH) and after phosphorus adsorption (CWH + P) was characterized to analyze its structural morphology and surface elemental composition. This was done using Scanning Electron Microscopy coupled with energy-dispersive X-ray Spectroscopy (SEM-EDX) on a JEOL-JSM 6490LV instrument equipped with a LaB6 filament, operating at an accelerating 20 kV.

### Fixed-bed adsorption studies

**Fig 1:**
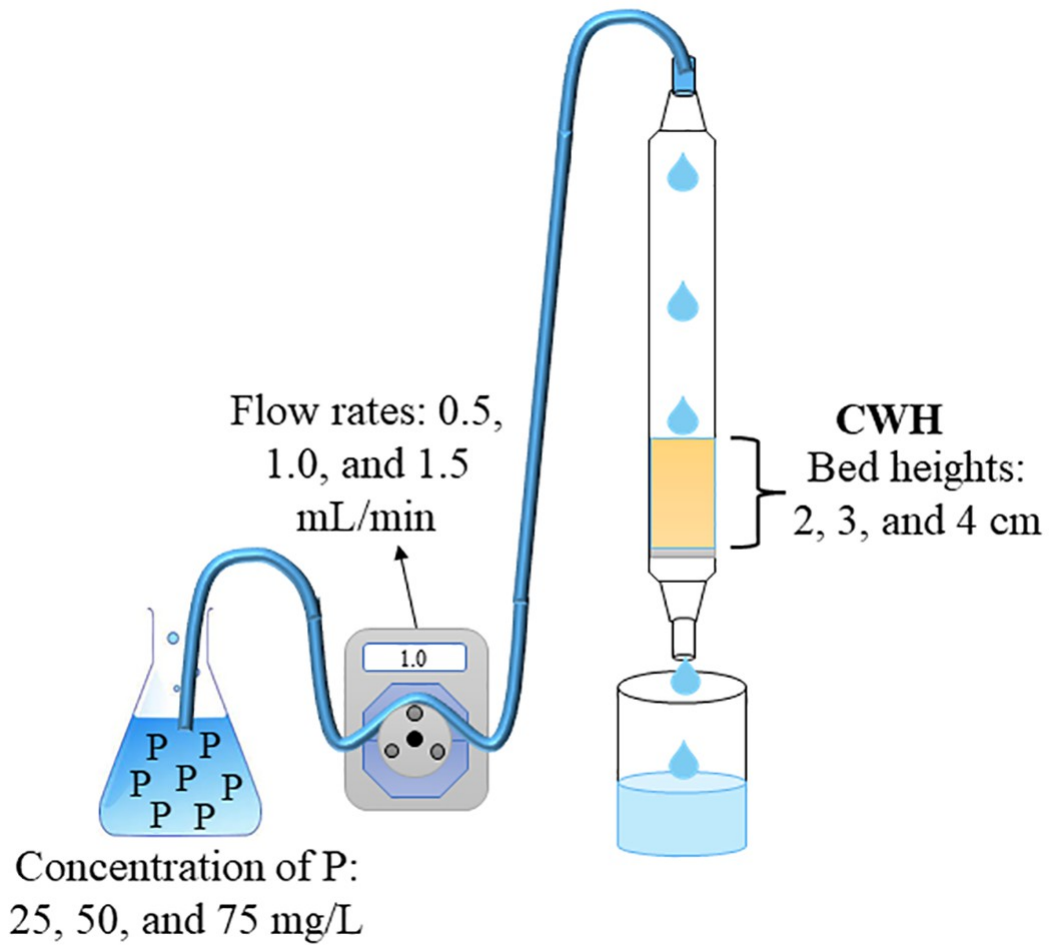
Graphic representation of the experiments in a fixed bed column.

Experiments were conducted on fixed bed columns to evaluate column performance under varied operating parameters, including bed volume, initial P concentration, and flow rate. The experiments were carried out in a glass column of 15 cm in height and 1.1 cm in inner diameter (See Fig 1). In brief, phosphate solutions with concentrations of 25, 50, and 75 mg/L (prepared from KH_2_PO_4_—Panreac, 99.0%) were pumped through the column using an adjustable peristaltic pump (Shenchen, LabV6-III), resulting in continuous flow rates of 0.5, 1.0, and 1.5 mL/min at different bed heights (2, 3, and 4 cm of CWH) (see S1 Table). Effluent samples were collected periodically and analyzed for residual phosphate concentration at different intervals of time. The concentration of P in the effluent was determined using the colorimetric method (Hach, PhosVer^®^ 3 Phosphate Reagent) with a DR 6000 spectrophotometer UV-VIS (Hach). The breakthrough curves at different experimental conditions were constructed by plotting the dimensionless concentration at the column outlet against time.

### Mathematical modeling of breakthrough curves

Breakthrough times and bed volumes play a crucial role in determining the dynamic operating conditions of an adsorption column [2, 3]. The overall performance of continuous flow adsorption columns is significantly influenced by the shape and length of the ion exchange zone that occurs during solid-liquid contact. This exchange zone progresses through the column section, gradually saturated with adsorbate, juxtaposed with the untouched adsorbent section. During the initial stage of the adsorption process, the top adsorbent layer of the column is "hit" by a high concentration of adsorbate; theoretically, this is where the greatest mass transfer occurs [31, 32]. Nevertheless, since the establishment of the mass transfer zone (MTZ) requires both time and column length, the initial behavior of the column cannot be considered reasonable, exhibiting a transient and unsteady-state regime. As the MTZ fully develops within the bed, its progress along the column becomes observable. At the end of the bed, the breakthrough curve reflects the shape of the MTZ [31]. The MTZ indicates the adsorbent efficiency, defined by the length of the adsorption zone in the column, as follows Eq (1):

$$\text{MTZ}=Z\left(\frac{t_{s}-t_{b}}{t_{s}}\right)$$ (1)

where, Z (cm) is the bed height, t_b_ and t_s_ (min) are the breakthrough time and the exhaustion time, respectively. The t_b_ is determined as the time when the P concentration exiting the column, C_t_, reaches 10% of the initial P concentration, C_0_, $\frac{{\text{C}}_{\text{t}}}{{\text{C}}_{0}}=0.1$. The t_s_ is the time when the P concentration exiting the column reaches 90% of the initial P concentration, $\frac{{\text{C}}_{\text{t}}}{{\text{C}}_{0}}=0.9$.

The total amount of P retained by the column, q_total_ (mg), at a flow rate, Q (mL/min), and a given P concentration is equivalent to the integral of P concentration in the fixed bed, C_ad_ (mg/L), respect to time, t (min), and it is calculated using Eq (2):

$$q_{total}=\frac{Q}{1000}\int_{t=0}^{t=total}C_{ad}dt$$ (2)

The maximum capacity of the column under steady-state conditions, q_eq_ (mg/g), is equal to the total amount of the P adsorbed (q_total_) per gram of adsorbent (w) at the end of the time, and it can be calculated according to Eq (3):

$$q_{eq}=\frac{q_{total}}{w}$$ (3)

This study employed four mathematical models, Thomas, Adams-Bohart, Yoon-Nelson, and Bed Depth Service Time, to characterize phosphorus (P) adsorption on CWH in a continuous system. These models are widely utilized in studying the column behavior of adsorbent-adsorbate systems [33], as they effectively describe breakthrough curves and provide parameters crucial for predicting the operational lifetime of a column during scale-up. Breakthrough curves, elucidated by these models, hold paramount importance in the design and operation of continuous adsorption systems.

#### Thomas model

The Thomas model assumes the adsorption process adheres to Langmuir isotherm with a pseudo-second-order rate expression. It postulates reversible reaction kinetics for the velocity driving forces devoid of axial dispersion within the adsorption column [33–35]. By fitting experimental data to the Thomas model, it becomes possible to ascertain the maximum adsorbate concentration in the solid phase, q_Th_, and determine the rate constant, k_Th_. The Thomas model [36], represented by Eq (4), can be expressed in its linear form as shown in Eq (5):

$$\frac{C_{t}}{C_{0}}=\frac{1}{1+exp\left(\frac{k_{Th}q_{Th}w}{Q}-k_{Th}C_{0}t\right)}$$ (4)

$$\text{ln}\;\left(\frac{C_{0}}{C_{t}}-1\right)=\frac{k_{Th}q_{Th}w}{Q}-k_{Th}C_{0}t$$ (5)

where C_0_ and C_t_ (mg/L) are the P initial and at time, t, concentration; k_Th_ (L/mg min) is the Thomas constant rate; q_Th_ (mg/g) is the adsorption capacity for CWH. Using a graph of $\text{ln}\;\left(\frac{{\text{C}}_{0}}{{\text{C}}_{\text{t}}}-1\right)$ vs t, values of k_Th_ and q_Th_, are calculated from the slope and the intercept, respectively.

#### Adams-Bohart model

This model primarily addresses the first region of the breakthrough curve ($\frac{{\text{C}}_{\text{t}}}{{\text{C}}_{0}}\leq 0.5$), operating under the assumption that equilibrium is not immediately achieved. It assumes a rectangular isotherm with a quasi-chemical rate expression, neglecting dispersive effects such as axial dispersion or mass transfer [35, 37, 38]. According to this model, the adsorption rate is directly proportional to the concentration of the adsorbed contaminant and the remaining capacity of the adsorbent. Consequently, the adsorption rate is favorable and is proportional to the residual adsorption capacity of the adsorbent and the concentration of adsorbed species [1, 34, 38, 39]. The Adams-Bohart model [40] equation and its linearized form are shown in Eqs (6) and (7), respectively:

$$\frac{C_{t}}{C_{0}}=exp\left(k_{BA}C_{0}t-\;k_{BA}N_{0}\frac{Z}{U_{0}}\right)$$ (6)

$$\text{ln}\;\left(\frac{C_{t}}{C_{0}}\right)=k_{BA}C_{0}t-k_{BA}N_{0}\frac{Z}{U_{0}}$$ (7)

where k_BA_ (L/mg min) is the Adams-Bohart constant rate; N_0_ (mg/L) is the column saturation concentration; U_0_ (cm/min) is the linear rate and it is calculated by dividing the flow rate (cm^3^/min) by the cross-sectional area of the column (cm^2^); values of k_BA_ and N_0_ are calculated from the slope and the intercept of Eq (7), respectively.

#### Yoon-Nelson model

The Yoon-Nelson model operates on a simple theoretical premise, obviating the need for data concerning the properties of the adsorbate or the specific type of adsorbent. The main assumption of this model is that the decrease in the adsorption rate of the adsorbate molecule is directly proportional to both the adsorption of the adsorbate and the breakthrough of the adsorbent [2, 33, 39, 41]. The Yoon-Nelson model [42] is shown in Eq (8), and the linear form can be expressed as follows in Eq (9):

$$\frac{C_{t}}{C_{0}}=\frac{exp\left(k_{YN}t-{\tau k}_{YN}\right)}{1+exp\left(k_{YN}t-{\tau k}_{YN}\right)}$$ (8)

$$\text{ln}\;\left(\frac{C_{t}}{C_{0}-C_{t}}\right)=k_{YN}t{-\tau k}_{YN}$$ (9)

where k_YN_ (L/mg min) is the Yoon-Nelson constant rate; τ (min) is the time required for 50% of the adsorbate breakthrough. Using a graph of $\text{ln}\;\left(\frac{{\text{C}}_{\text{t}}}{{\text{C}}_{0}-{\text{C}}_{\text{t}}}\right)$ vs, t, values of k_YN_ and τ can be determined using the slope and the intercept, respectively.

#### Bed Depth Service Time model

The Bed Depth Service Time model (BDST) is a predictive tool for estimating the packed bed material’s duration within the column before requiring regeneration or replacement [43]. It operates under the assumption that the adsorption rate is maintained due to surface reactions between the adsorbate and the unused capacity of the adsorbent, with negligible intraparticle diffusion [2, 33, 41]. This indicates that equilibrium within the bed is not instantaneous, thereby rendering the rate of the adsorption process proportional to the remaining adsorption capacity within the medium [38].

The constants derived from the BDST model are invaluable for scaling the process across different flow rates and concentrations without requiring additional experimentation. It can be applied to predict column performance for any given bed height if values for certain heights are known. The model establishes a linear relationship between the bed height (Z) and the service time (t) of the column [41]. This linear relationship between bed depth (i.e., height) and service time is expressed by Eq (10):

$$\text{t}=\frac{N_{0}Z}{C_{0}U_{0}}-\frac{1}{k_{BDST}}\;\text{ln}\;\left(\frac{C_{0}}{C_{t}}-1\right)$$ (10)

where k_BDST_ (L/mg min) is the rate constant of the BDST model; N_0_ (mg/L) is the adsorption capacity; and t is the service time (min). Values of N_0_ and k_BDST_ can be determined from the slope and the intercept of Eq (10), respectively.

### Municipal wastewater application

To evaluate the column’s effectiveness in treating real wastewater, we conducted a column adsorption experiment using simulated municipal wastewater prepared according to OECD guidelines (2001) [44, 45] (see S2 Table). The column was packed with 0.9 g of CWH, and the wastewater sample was pumped using a peristaltic pump at a controlled flow rate of 1.0 mL/min. Effluents from the column were collected and analyzed at different periods. The concentration of P remaining in the solution was determined by the colorimetric method (Hach, PhosVer^®^ 3 Phosphate Reagent) using a DR 6000 spectrophotometer UV-VIS (Hach).

## Results and discussions

### Fixed bed column studies—Effect of flow rate, initial P concentration, and bed height variations

**Fig 2:**
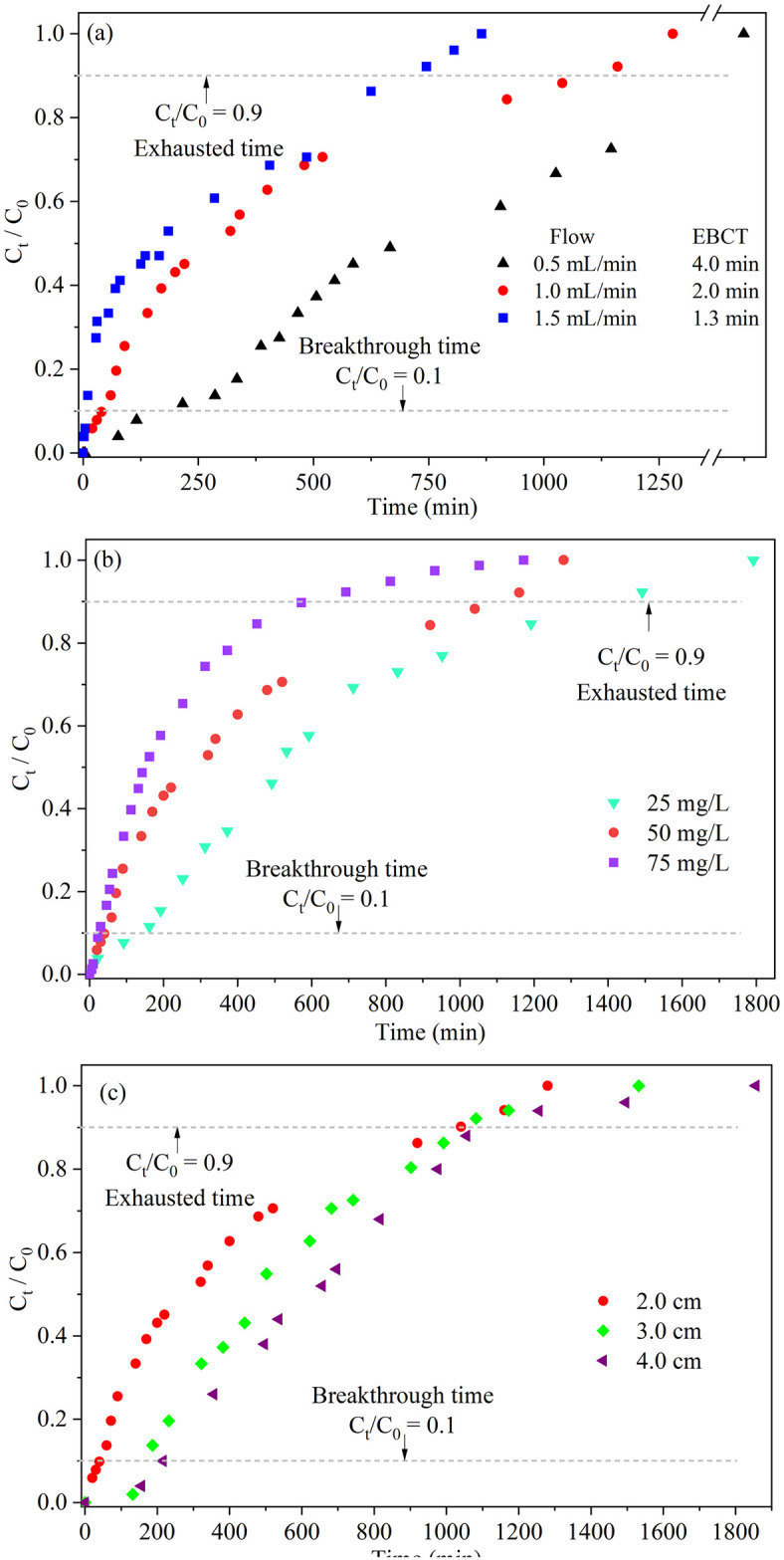
Breakthrough curves varying (a) flow rate, (b) initial P concentration, and (c) bed height of P adsorption on CWH. (a) C0 = 50 mg/L, bed height = 2 cm; (b) flow = 1.0 mL/min, bed height = 2 cm; (c) C0 = 50 mg/L, flow = 1.0 mL/min. EBCT: empty bed contact time, Ct: P concentration on the time, C0: P initial concentration.

Fig 2a–2c shows the effect of varying flow rate, initial P concentration, and bed height on the breakthrough curves of P adsorption on CWH. As the flow rate increases, the breakthrough time (t_b_) and exhausted time (t_s_) decrease. Specifically, according to Table 3, when the flow rate increased from 0.5 to 1.5 mL/min, t_b_ decreased from 171 to 7.15 min, and t_s_ decreased from 1786.80 to 701 min. This observation indicates that there is increased contact time between P and CWH at a lower flow rate, leading to a higher probability of interaction with the adsorption active sites. Hence, the flow rate emerges as a crucial parameter in fixed bed columns, given its direct influence on contact time and mass transfer rate.

Similarly, when the initial P concentration increases from 25 to 75 mg/L, a comparable trend is observed with t_b_ and t_s_. The time taken for the column to reach 10% saturation decreases from 134 to 25.2 min, while the time to reach 90% saturation decreases from 1402 to 584 min. This indicates that the column saturates approximately 2.4 times faster at a higher concentration of 75 mg/L than 25 mg/L. This acceleration in saturation can be attributed to the rapid occupation of available active sites at higher concentrations [2]. Conversely, as the bed height increases from 2.0 to 4.0 cm, there is an observed increase in t_b_ from 41 to 216 min, and t_s_ also increases from 1034 to 1122.67 min. This phenomenon can be attributed to the higher bed volume, which offers increased active binding sites within the CWH to capture P incoming P within the column [2, 32]. Similar studies have linked this behavior to the relationship between bed height and adsorbent surface area, providing more binding sites for adsorbate adsorption [1, 2, 41].

Table 1 shows that when the flow rate varies from 0.5 to 1.5 mL/min and the initial P concentration from 25 to 75 mg/L, the MTZ changes slightly between 1.81 and 1.98 cm and 1.81 and 1.92 cm, respectively. However, when increasing the bed height from 2 to 4 cm, a notable increase in MTZ is observed, rising from 1.92 to 3.23 cm. This indicates that the partial saturation zone moves through the column in the flow direction determined by the adsorbate concentration, adsorbent capacity, and flow rate [41]. From these results, the column can operate until MTZ has reached the end of the column. Before MTZ reaches the end of the column, the effluent is practically free of adsorbate, but when MTZ reaches the end of the column, the P concentration in the effluent starts to increase gradually. The above agrees with the behavior described by Yoon and Nelson (1984) and Patel (2019) [33, 41].

**Table 1:** Experimental breakthrough parameters.

Flow (mL/min)	C_o_ (mg/L)	Bed height (cm)	t_b_ (min)	t_s_ (min)	MTZ (cm)	q_total_ (mg)	q_eq_ (mg/g)
**0.5**	**50.00**	**2.0**	171.00	1786.80	1.81	22.84	26.55
**1.0**			41.00	1034.00	1.92	20.39	23.70
**1.5**			7.15	701.00	1.98	20.80	23.64
**1.0**	**25.00**		134.00	1402.00	1.81	16.34	18.56
	**75.00**		25.20	584.00	1.91	18.79	21.35
	**50.00**	**3.0**	169.58	1065.33	2.52	28.49	19.65
		**4.0**	216.00	1122.67	3.23	33.01	15.87

Table 1 illustrates the impact of varying flow rates on the column’s adsorption capacity for P. With an increase in flow rate from 0.5 to 1.5 mL/min, the maximum P adsorption capacity decreased slightly from 22.84 to 20.80 mg/g (~8%). This reduction can be attributed to the shorter residence times of the P solution within the bed column and the heightened turbulence experienced at higher flow rates. A lower flow rate predominates the mass transport of P over intra-particle diffusion, causing insufficient contact time between adsorbate and adsorbent and resulting in weaker interactions. Consequently, P exits the column before reaching the adsorption equilibrium.

Moreover, the accelerated downward movement of the primary adsorption zone in the fixed bed, induced by increased flow rates, further contributes to the decreased adsorption capacity of the column [46]. Conversely, low flow rates ensure prolonged contact time, facilitating stronger adsorbate-adsorbent interactions [3]. It is also observed that greater bed heights extend the residence time between P and CWH. As the bed height increased from 2 to 4 cm of exhausted CWH material, the time required for C_t_/C_0_ = 10% (t_b_) increased from 41 min to 216 min. This finding aligns with the study conducted by Manjunath and Kumar (2021) [47], highlighting that larger bed heights diminish the mass transfer rate by increasing the contact time between the adsorbate and adsorbent.

**Fig 3:**
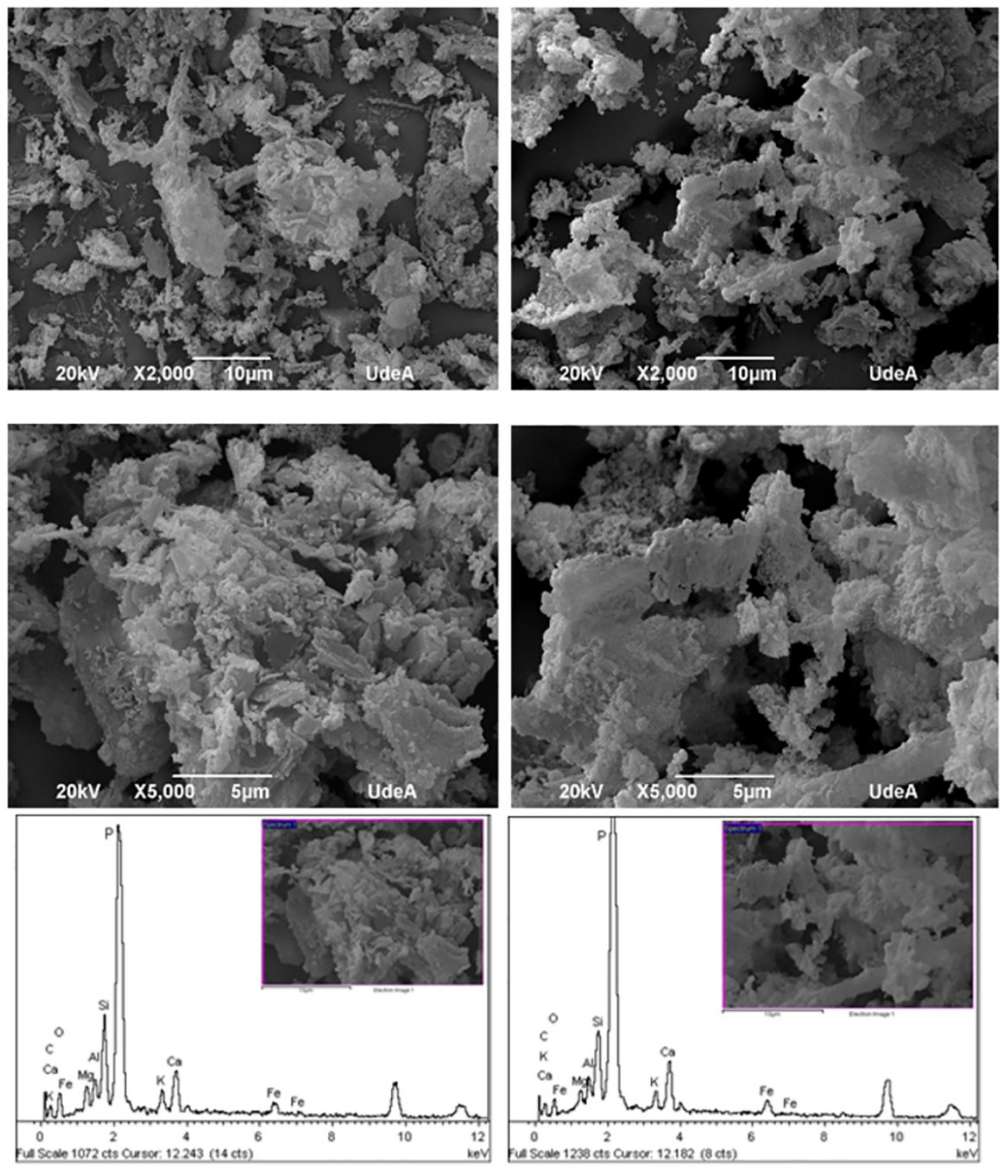
SEM micrographs of CWH before (left side) and after P adsorption (right side).

To evaluate the morphology and surface elemental composition, the material was characterized before (CWH) and after phosphorus adsorption (CWH + P). Fig 3 shows the SEM micrographs of CWH and CWH + P, where the materials exhibit an irregular morphology with a rough, porous surface. The surface is primarily composed of elements such as C, Ca, O, K, Fe, Mg, Al, Si, and P, with a noticeable increase in phosphorus intensity in the CWH + P sample, indicating higher P concentration on the surface after adsorption.

The presence of Ca in the material is particularly significant, as calcium plays a crucial role in phosphorus adsorption by promoting the formation of calcium-phosphate complexes. Additionally, the SEM images reveal swollen surfaces, with pores formed as volatile matter is released during heating, expanding the microstructure of the biomass. The resulting CWH retains its porous surface area in CWH + P, which may enhance the soil’s water retention capacity and improve its efficiency as a fertilizer [22]. This is particularly inspiring as it suggests that the material could play a key role in fostering the growth of beneficial microorganisms and retaining essential nutrients, thereby significantly improving soil quality.

### Modeling of breakthrough curves

Table 2 and S1a–S1g Fig provide parameters for the Thomas, Adams-Bohart, and Yoon-Nelson models. The experimental data present a good fit to these models, with correlation coefficients, R^2^, up to 0.98, indicating that these models can represent the experimental values.

**Table 2:** Thomas, Adams-Bohart, and Yoon-Nelson models parameters for the adsorption of P on CWH.

**Flow (mL/min)**	**0.5**	**1.0**	**1.5**	**1.0**		**1.0**	
**C**_**o**_ **(mg/L)**	**50.00**			**25.00**	**75.00**	**50.00**	
**Height (cm)**	**2.0**			**2.0**		**3.0**	**4.0**
**Thomas Model**							
**k**_**Th**_ **x 10**^**−3**^ **(L/mg min)**	0.06	0.07	0.09	0.14	0.07	0.08	0.09
**q**_**Th**_ **(mg/g)**	23.91	20.66	18.22	18.47	20.45	18.60	16.07
**R** ^**2**^	0.91	0.94	0.94	0.92	0.93	0.98	0.96
**Bohart-Adams Model**							
**k**_**BA**_ **x 10**^**−3**^ **(L/mg min)**	0.09	0.24	1.30	0.25	0.24	0.10	0.12
**N**_**o**_ **(mg/L)**	9861.64	5697.41	1873.41	6402.18	5483.42	9929.49	8566.88
**R** ^**2**^	0.96	0.92	0.92	0.96	0.96	0.96	0.92
**Yoon-Nelson Model**							
**k**_**YN**_ **x 10**^**−3**^ **(1/min)**	3.00	3.50	4.80	3.70	5.60	4.20	4.50
**τ (min)**	806.47	348.31	209.56	625.03	230.75	528.88	668.49
**R** ^**2**^	0.90	0.94	0.94	0.92	0.93	0.98	0.96

#### Thomas model

The Thomas model demonstrates a strong fit to the experimental data, as depicted in S1a–S1g Fig. In Table 2, the values of q_Th_ and k_Th_ exhibit contrasting trends with the increase in flow rate from 0.5 to 1.5 mL/min. While q_Th_ decreases from 23.91 to 18.22 mg/g, k_Th_ increases from 0.06 to 0.09 L/mg min. At higher flow rates, the weakening of mass driving force leads to rapid adsorption of P onto active sites, shortening the contact time between adsorbent and adsorbate. This results in an incomplete adsorption process and, consequently, lower adsorption capacity. Conversely, when the initial P concentration rises from 25 to 75 mg/L, q_Th_ increases from 18.47 to 20.45 mg/g, while k_Th_ decreases from 0.14 to 0.07 L/mg min. This is attributed to the heightened resistance to mass transfer caused by higher P concentration, which decreases the velocity constant [2]. Furthermore, Table 2 shows that with an increase in bed depth from 2 to 4 cm at a constant flow rate of 1 mL/min, the adsorption capacity (q_Th_) decreases from 20.66 to 16.07 mg/g, while the constant rate (k_Th_) increases from 0.07 to 0.09 L/mg min. This behavior aligns with findings by Lv and Li (2023), suggesting that non-adsorbed phosphate remaining in the adsorption layer induces axial diffusion, while the Thomas model is ideally obtained without axial diffusion [4]. These observations are consistent with those reported by other studies [3, 4, 29]. Notably, the calculated values of q_Th_ using this model align with the experimental values of q_eq_ presented in Table 1.

#### Adams-Bohart model

The Adams-Bohart model effectively fits the initial segment of the breakthrough curve (C_t_/C_0_ < 0.5). This is a characteristic consistent with its typical application in describing the first 50% of the breakthrough curve, as observed in S1 Fig and corroborated by previous studies [1, 3, 48]. Examining Table 2 reveals that with an increase in flow rate from 0.5 to 1.5 mL/min, the mass transfer coefficient (k_BA_) increases from 0.09 to 1.30 L/mg min. At the same time, the column’s saturation concentration (N_0_) decreases from 9861.64 to 1873.41 mg/L. This suggests that during the initial part of adsorption on the column, external mass transfer predominantly governs the global kinetics of the system. On the other hand, by increasing the bed height from 2 to 4 cm, k_BA_ decreases from 0.24 to 0.12 L/mg min, and N_0_ increases from 5697.41 to 8566.88 mg/L. These trends are consistent with findings from other research studies [1, 48], underscoring these behavior’s reliability and reproducibility across different experimental setups.

#### Yoon-Nelson model

According to the Yoon-Nelson model, the rate constant (k_YN_) and the time required to reach 50% breakthrough time (τ) were determined (Table 2). As the flow rate increased from 0.5 to 1.5 mL/min, τ decreased from 806.47 to 209.56 min, while k_YN_ increased from 3.00 to 4.80 1/min. Similarly, as the initial P concentration rose from 25 to 75 mg/L, τ decreased from 625.03 to 230.75 min, while k_YN_ increased from 3.70 to 5.60 1/min. These changes are attributed to the heightened mass driving force from the liquid phase to the solid-liquid interface. This results in increased P mobility and occupation of more CWH active sites. Consequently, the mass transfer zone moves faster, reducing the time required to achieve a 50% breakthrough (τ) and increasing k_YN_ [4, 47]. This phenomenon indicates that CWH saturation on the column occurs more rapidly at higher flow rates [4, 9, 49]. Notably, a higher τ value reflects better column performance, as reported by Omitola et al. (2002) [50]. On the other hand, increasing the bed height from 2 to 4 cm results in a slight increase in k_YN_ from 3.50 to 4.50 1/min and an increase in τ from 348.31 to 668.49 min. This is attributed to the greater depth of the bed, which increases resistance to mass transfer and extends the contact time between P and CWH [4, 47, 49].

#### Bed Depth Service Time model

The BDST model is widely used in designing and operating fixed bed columns for wastewater treatment systems to gauge the longevity of the adsorbent bed before regeneration or replacement becomes necessary [2, 43, 51].

**Fig 4:**
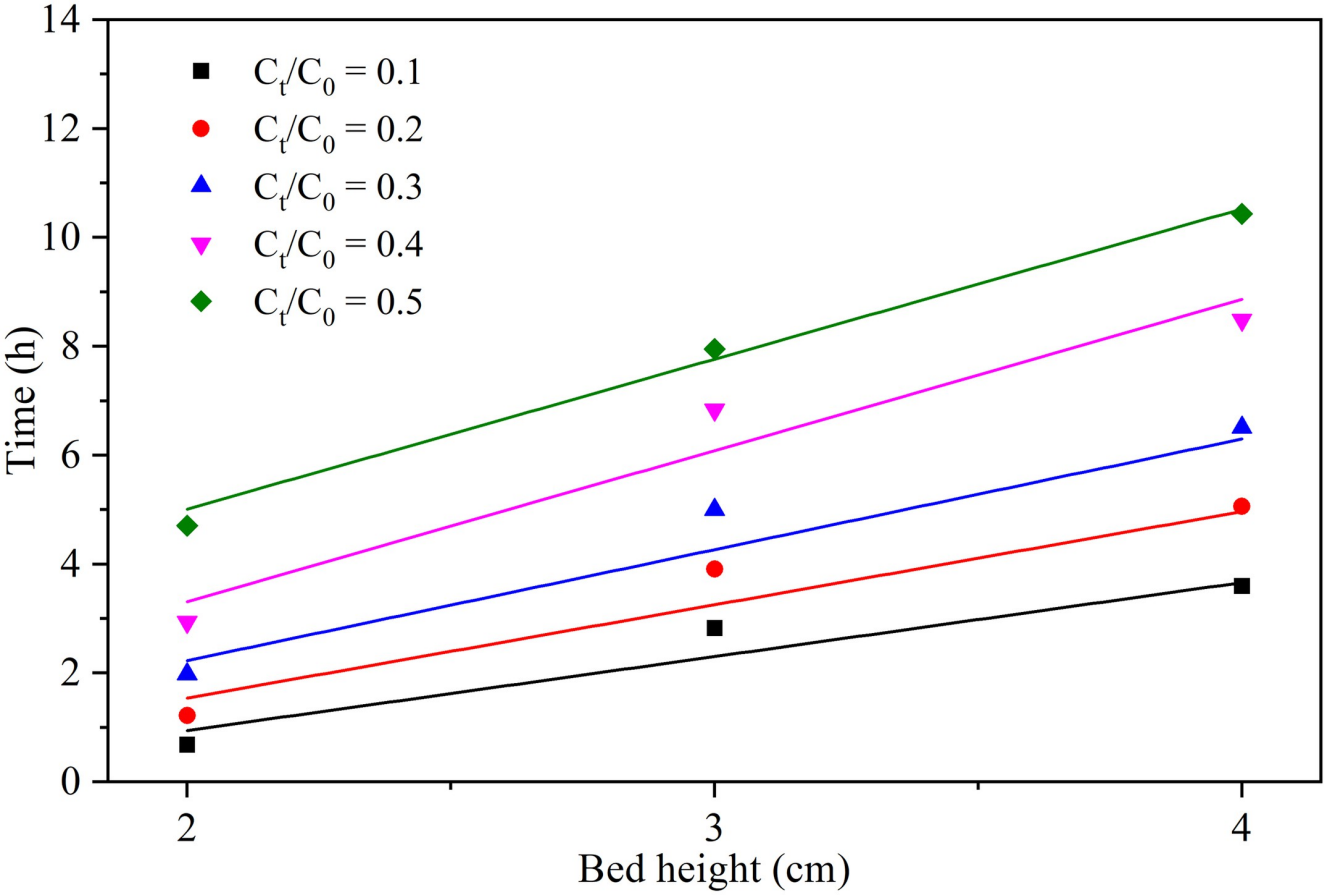
Bed Depth Service Time (BDST) model for Ct/C0 from 0.1 to 0.5 breakthrough at different bed depths (2, 3, and 4 cm), flow rate (1.0 mL/min), and constant influent concentration (50 mg/L). Ct: P concentration on the time, C0: P initial concentration.

In this work, we employed the BDST model to investigate the effect of bed height on removing P from aqueous solutions using CWH in fixed bed columns. The experiments were conducted using various breakthrough points, ranging from C_t_/C_0_ = 0.1, 0.2, 0.3, 0.4, and 0.5 and bed depths of 2, 3, and 4 cm (see Fig 4). The parameters obtained by fitting the experimental data of the BDST model are summarized in Table 3. Notably, the analysis reveals a robust linear correlation (R^2^ ≥ 0.93), indicating that the BDST model effectively characterizes the experimental values of P adsorption on CWH.

**Table 3:** BDST model parameters of P adsorption on CWH.

BDST model						
**Bed height (cm)**	**C**_**t**_ **/C**_**0**_	**0.1**	**0.2**	**0.3**	**0.4**	**0.5**
	**N**_**o**_ **(mg/L)**	4603.66	6058.41	7162.24	8769.04	9036.32
**2**	**k**_**BDST**_ **x10**^**-5**^**(L/mg min)**	36.57	19.60	12.35	6.02	0.01
**3**	**k**_**BDST**_ **x10**^**-5**^**(L/mg min)**	41.48	19.63	12.53	6.03	0.01
**4**	**k**_**BDST**_ **x10**^**-5**^**(L/mg min)**	36.53	20.86	10.96	6.03	0.01
	**R** ^**2**^	0.93	0.95	0.96	0.95	0.99
	**Z**_**0**_ **(cm)**	1.37	1.23	1.02	0.81	0.31

From Table 3, the adsorption capacity (N_0_) increases from 4603.66 to 9036.32 mg/L with the increase of C_t_/C_0_ from 0.1 to 0.5. This may be attributed to the fact that P occupies more active sites on CWH at higher values of C_t_/C_0_, which improves adsorption capacity [43].

By setting t = 0 and solving Eq (10) for Z_0_, produces the following Eq (11) [1]:

$$Z_{0}=\frac{U_{0}}{k_{BDST}C_{0}}ln\left(\frac{C_{0}}{C_{t}}-1\right)$$ (11)

where Z_0_ (cm) is the critical bed height, which is the minimum bed height required to obtain the desired effluent concentration (C_t_).

Table 3 reveals a decreasing trend in Z_0_, ranging from 1.37 to 0.31 cm as C_t_/C_0_ increases from 0.1 to 0.5. This indicates that the minimum bed depth required to achieve the desired effluent concentration (C_t_) diminishes as the CWH column allows more P to pass through.

These findings underscore the significance of flow rate and bed height in effectively utilizing CWH as a fixed bed column adsorbent for P removal from water. Overall, these results highlight the utility of the BDST model in offering insights into the performance of large-scale wastewater treatment systems aimed at P removal using CWH filled fixed bed columns [43].

#### Comparison of breakthrough models

The Thomas, Adams-Bohart, and Yoon-Nelson models proved to be effective in accurately describing the breakthrough curves observed during P adsorption on CWH, enabling the determination of key column properties essential for process design. Notably, the Thomas model demonstrated its efficacy by observing low values of Δq (q_Th_ − q_eq_), ranging between 0.1 and 5.42 (See Table 4). These results suggest that maintaining high bed adsorbent levels alongside moderate flow rates enhances column efficiency in P removal over prolonged operational periods. Additionally, the BDST model exhibited a good linear relationship between the amount of CWH employed and the service time, thereby facilitating the determination of the requisite amount of adsorbent needed to attain a specified breakthrough criterion. This behavior underscores the feasibility of implementing the fixed bed column within a continuous-flow wastewater system.

**Table 4:** Thomas adsorption capacity and maximum capacity of the column for adsorption of P on CWH.

Flow (mL/min)	C_o_ (mg/L)	Bed height (cm)	q_Th_ (mg/g)	q_eq_ (mg/g)	Δq (q_Th_ − q_eq_)
**0.5**	**50.0**	**2.0**	23.91	26.55	2.64
**1.0**			20.66	23.70	3.05
**1.5**			18.22	23.64	5.42
**1.0**	**25.0**		18.47	18.56	0.10
	**75.0**		20.45	21.35	0.90
	**50.0**	**3.0**	18.62	19.65	1.04
		**4.0**	16.07	15.87	0.20

### Municipal wastewater application

**Fig 5:**
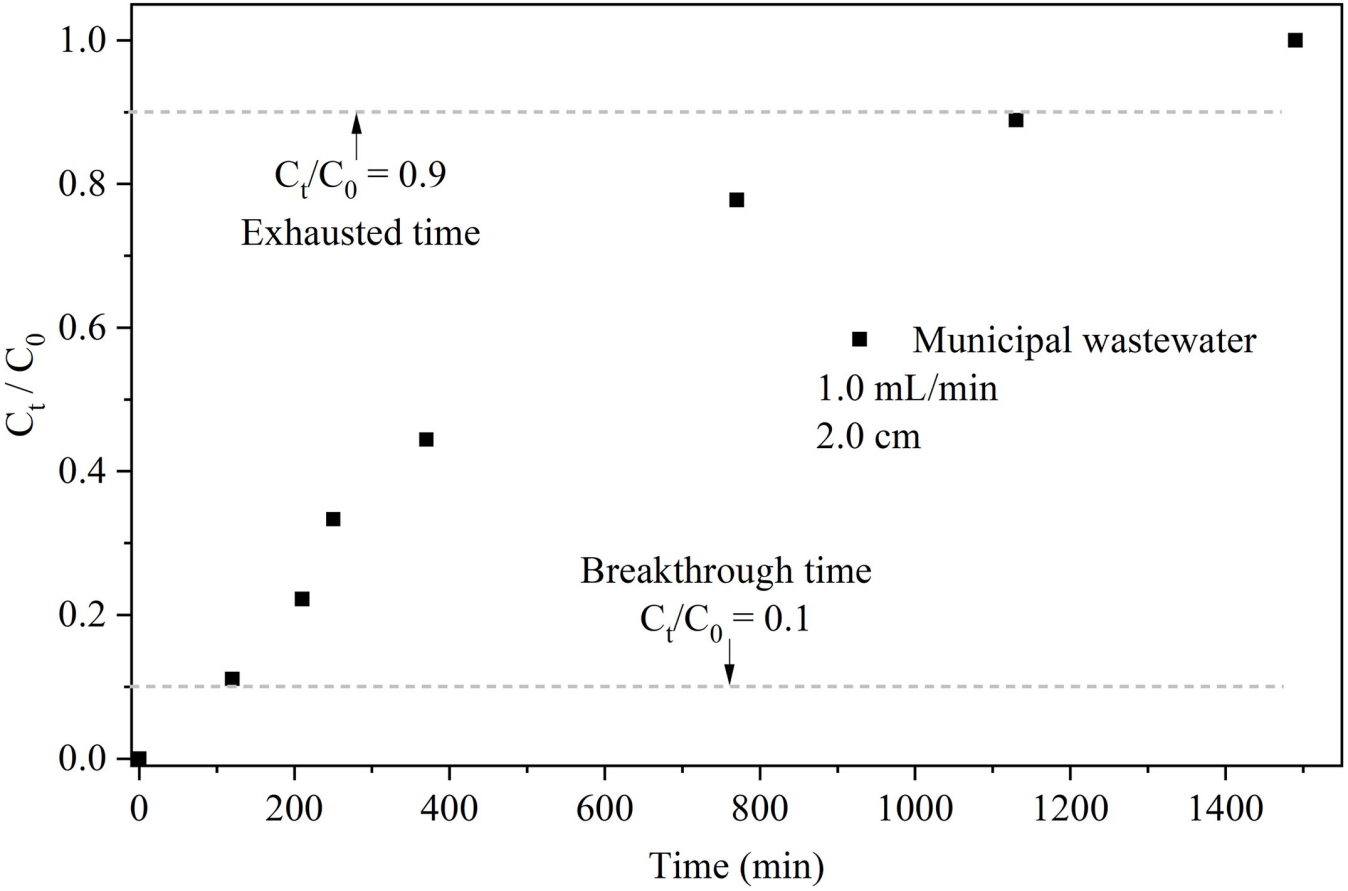
Column adsorption using synthetic municipal wastewater. C0 = 9 mg/L, flow = 1.0 mL/min, and bed height = 2.0 cm. Ct: P concentration on the time, C0: P initial concentration.

A packed column was employed to assess CWH’s efficacy in removing P from municipal wastewater. Fig 5 illustrates the breakthrough curve depicting P adsorption on CWH using synthetic municipal wastewater, in while Table 5 shows the experimental breakthrough parameters. Notably, t_b_ and t_s_ are 108 min and 1166 min, respectively, with a maximum adsorption capacity of P on the column of 5.20 mg/g.

**Table 5:** Experimental breakthrough parameters to municipal wastewater.

Flow (mL/min)	Bed height, Z (cm)	t_b_ (min)	t_s_ (min)	MTZ (cm)	q_total_ (mg)	q_eq_ (mg/g)
**1.0**	**2.0**	108.00	1166.00	1.81	4.74	5.20

The findings presented above underscore the suitability of a CWH-packed column for continuous P adsorption experiments without compromising the material’s active phase. Table 6 compares phosphorus adsorption capacities determined by the Thomas model across various adsorbent materials. Water hyacinth (CWH) demonstrates a notably higher Thomas adsorption capacity for phosphorus than other materials derived from physical and chemical treatments in continuous systems, highlighting a distinct advantage of CWH over alternative adsorbents. Additionally, this table now emphasizes not only the effectiveness of water hyacinth for phosphorus removal but also its potential for removing heavy metals, dyes, and pesticides. These additions further underscore the versatility and significance of water hyacinth as an adsorbent material for addressing a wide range of environmental pollutants.

**Table 6:** Comparison of Thomas adsorption capacity to several adsorbents for removal of P and other pollutants.

Adsorbent	Flow (mL/min)	Bed height (cm)	q_Th_ (mg/g)	References
**Calcinated Water Hyacinth—(P)**	1.0	2	23.91	This study
**PBP immobilized on a resin material—(P)**	0.2	3	13.10	[2]
**Sewage by Zr(IV)-loaded okara—(P)**	12	23	12.21	[1]
**Calcined Mg**_**3**_ **–Fe layered double hydroxides—(P)**	0.0004	12	10.25	[41]
**Ca-modified attapulgite—(P)**	1.0	4	13.76	[4]
**Leftover coal—(P)**	1.0	6	0.25	[29]
**Water hyacinth biochar (WHBC) and ferrihydrite (FH), FH/WHBC—(Glyphosate)**	5	-	99.80	[52]
**Water hyacinth–powder—(Cr(III))**	0.5	25	2.95	[53]
**Co-modified water hyacinth biochar with Fe and Mg, Fe/Mg–WHBC—(Imidacloprid)**	3.5	-	34.90	[54]
**Water hyacinth activated with KOH, ABW—(Methylene blue)**	10	4	362.66	[55]

## Conclusions

In conclusion, this study marks a significant advancement in applying calcined water hyacinth (CWH) as an adsorbent material for phosphorus (P) removal in continuous-flow systems. Our investigation provides a comprehensive evaluation of how key operational parameters—specifically influent flow rate and adsorbent height—impact the system’s adsorption performance and breakthrough characteristics. These findings have significant implications for the fields of environmental engineering, water treatment, and sustainable agriculture, as they offer a potential solution for phosphorus removal and soil enrichment.

The results demonstrate that increased flow rates accelerate the saturation of the CWH surface with phosphorus, reducing both breakthrough and exhaustion times. Specifically, we observed an 8.93% decrease in the maximum adsorption capacity of the material as flow rates increased. This finding indicates that higher flow rates enhance throughput and result in quicker exhaustion of the adsorbent, necessitating careful optimization to balance efficiency and capacity.

Conversely, increasing the adsorbent height within the fixed-bed column significantly improved performance by extending breakthrough and exhaustion times. This enhancement suggests that a larger adsorbent volume provides increased surface area and longer contact time between phosphorus and the adsorbent, ultimately boosting overall adsorption efficiency and prolonging the effective life of the column.

When applied to synthetic municipal wastewater containing 9 mg/L phosphorus, the continuous-flow system achieved a notable maximum column capacity (q_eq_) of 5.20 mg/g. This result underscores the substantial potential of CWH as an effective and sustainable solution for phosphorus removal, highlighting its applicability in real-world water treatment scenarios where efficient and cost-effective solutions are paramount.

Moreover, the study reveals an additional benefit of the exhausted CWH material, which can be repurposed as a soil amendment or fertilizer feedstock. This exciting finding opens up new possibilities for the use of CWH, as previous research has demonstrated the bioavailability of the adsorbed phosphorus in these materials, suggesting that the spent adsorbent could contribute to nutrient recycling and soil enrichment without the need for further reusability studies. This potential for soil enrichment should excite the audience about the additional benefits of the research.

Looking ahead, future research should focus on refining and optimizing the operational conditions of the continuous-flow system to enhance its performance and cost-efficiency. This includes exploring various scaling processes, improving the economic feasibility of large-scale applications, and investigating the long-term stability and effectiveness of the adsorbent under diverse operational conditions. By addressing these areas, the practical implementation of CWH in phosphorus removal technologies can be further advanced, underscoring the urgency and importance of your work in contributing to more sustainable water treatment solutions.

## Supporting information

S1 Table Continuous flow fixed bed column experimental design. (DOCX)

S2 Table Chemical composition of municipal wastewater. (DOCX)

S1 Fig Breakthrough curves modeling by Thomas, Adams-Bohart, and Yoon-Nelson models for the adsorption of P on CWH. (DOCX)

S1 Data (XLSX)
