# Supplementary material for: Effective phosphorus removal using transformed water hyacinth: Performance evaluation in fixed-bed columns and practical applications
Source: PLoS One. 2024 Nov 21;19(11):e0312432. doi: 10.1371/journal.pone.0312432 (PMC11581350; doi:10.1371/journal.pone.0312432)
Supplement: S1 Table — (DOCX) [file pone.0312432.s001.docx]

**Supporting information**

Effective phosphorus removal using transformed water hyacinth: Performance evaluation in fixed-bed columns and practical applications

Anyi Ramirez-Muñoz^a b^, Elizabeth Flórez^a^*, Raúl Ocampo-Perez^c^, and Nancy Acelas^a^*

^a^Grupo de investigación Materiales con Impacto (Mat&mpac), Facultad de Ciencias Básicas, Universidad de Medellín, Carrera 87 No. 30-65, Medellín 050026, Colombia

^b^ Laboratorio Nacional de Proyección Térmica (CENAPROT), Centro de Investigación y de Estudios Avanzados Del IPN, Libramiento Norponiente 2000 Fracc. Real de Juriquilla, 76230, Querétaro, México

^c^Centro de Investigación y de Estudios de Posgrado, Facultad de Ciencias Químicas, Universidad Autónoma de San Luis Potosí, 78260, San Luis Potosí, México

^*^Corresponding author

E-mail address: nyacelas@udemedellin.edu.co; elflorez@udemedellin.edu.co

Chemical reagents used:

KH_2_PO_4_ was obtained from Panreac. PhosVer® 3 Phosphate Reagent was obtained from Hach. Sodium chloride (NaCl), potassium monohydrogen phosphate (K_2_HPO_4_), peptone, meat extract, urea (CH_4_N_2_O), magnesium sulfate (MgSO_4_.7H_2_O), calcium chloride (CaCl_2_.2H_2_O), and sodium bicarbonate (NaHCO_3_) were obtained from Merck.

**Table S1. Continuous flow fixed bed column experimental design**.

| **Flow (mL/min)** | **Bed height (cm)** | **Initial concentration (mg/L)** | **Adsorbent amount (g)** | **Bed Volume, BV (mL)** | **EBCT (min)** |
| --- | --- | --- | --- | --- | --- |
| **0.5** | 2.0 | 50 | 0.9 | 2.0 | 4.0 |
| **1.0** | 2.0 | 50 | 0.9 | 2.0 | 2.0 |
| **1.5** | 2.0 | 50 | 0.9 | 2.0 | 1.3 |
| **1.0** | 2.0 | 25 | 0.9 | 2.0 | 2.0 |
| **1.0** | 2.0 | 75 | 0.9 | 2.0 | 2.0 |
| **1.0** | 3.0 | 50 | 1.5 | 3.0 | 3.0 |
| **1.0** | 4.0 | 50 | 2.1 | 4.0 | 4.0 |
