# Supplementary material for: Effective phosphorus removal using transformed water hyacinth: Performance evaluation in fixed-bed columns and practical applications
Source: PLoS One. 2024 Nov 21;19(11):e0312432. doi: 10.1371/journal.pone.0312432 (PMC11581350; doi:10.1371/journal.pone.0312432)
Supplement: S2 Table — (DOCX) [file pone.0312432.s002.docx]

**Supporting information**

Effective phosphorus removal using transformed water hyacinth: Performance evaluation in fixed-bed columns and practical applications

Anyi Ramirez-Muñoz^a b^, Elizabeth Flórez^a^*, Raúl Ocampo-Perez^c^, and Nancy Acelas^a^*

^a^Grupo de investigación Materiales con Impacto (Mat&mpac), Facultad de Ciencias Básicas, Universidad de Medellín, Carrera 87 No. 30-65, Medellín 050026, Colombia

^b^ Laboratorio Nacional de Proyección Térmica (CENAPROT), Centro de Investigación y de Estudios Avanzados Del IPN, Libramiento Norponiente 2000 Fracc. Real de Juriquilla, 76230, Querétaro, México

^c^Centro de Investigación y de Estudios de Posgrado, Facultad de Ciencias Químicas, Universidad Autónoma de San Luis Potosí, 78260, San Luis Potosí, México

^*^Corresponding author

E-mail address: nyacelas@udemedellin.edu.co; elflorez@udemedellin.edu.co

**Table S2. Chemical composition of municipal wastewater.**

| **Reagents** | **Concentration mg/L** |
| --- | --- |
| **Peptone** | 100 |
| **Meat extract** | 110 |
| **Urea** | 30 |
| **K_2_HPO_4_** | 28 |
| **MgSO_4_.7H_2_O** | 2 |
| **CaCl_2_.2H_2_O** | 40 |
| **NaCl** | 7 |
| NaHCO_3_ | 96 |
