# Supplementary material for: Effective phosphorus removal using transformed water hyacinth: Performance evaluation in fixed-bed columns and practical applications
Source: PLoS One. 2024 Nov 21;19(11):e0312432. doi: 10.1371/journal.pone.0312432 (PMC11581350; doi:10.1371/journal.pone.0312432)
Supplement: S1 Fig — (DOCX) [file pone.0312432.s003.docx]

**Supporting information**

Effective phosphorus removal using transformed water hyacinth: Performance evaluation in fixed-bed columns and practical applications

Anyi Ramirez-Muñoz^a b^, Elizabeth Flórez^a^*, Raúl Ocampo-Perez^c^, and Nancy Acelas^a^*

^a^Grupo de investigación Materiales con Impacto (Mat&mpac), Facultad de Ciencias Básicas, Universidad de Medellín, Carrera 87 No. 30-65, Medellín 050026, Colombia

^b^ Laboratorio Nacional de Proyección Térmica (CENAPROT), Centro de Investigación y de Estudios Avanzados Del IPN, Libramiento Norponiente 2000 Fracc. Real de Juriquilla, 76230, Querétaro, México

^c^Centro de Investigación y de Estudios de Posgrado, Facultad de Ciencias Químicas, Universidad Autónoma de San Luis Potosí, 78260, San Luis Potosí, México

^*^Corresponding author

E-mail address: nyacelas@udemedellin.edu.co; elflorez@udemedellin.edu.co

| 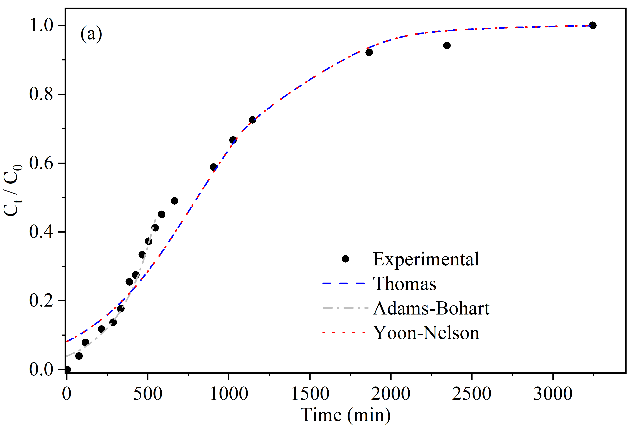 | 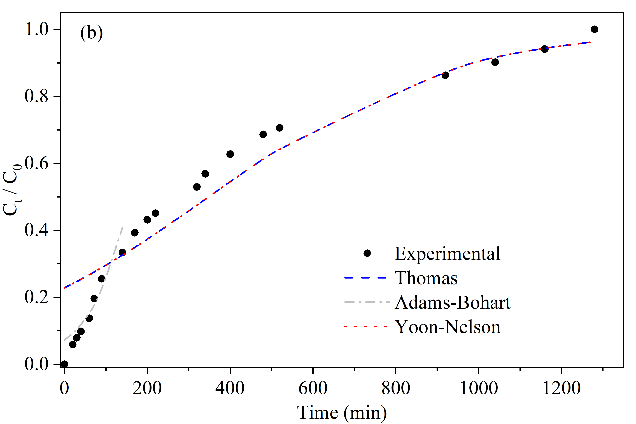 |
| --- | --- |
| 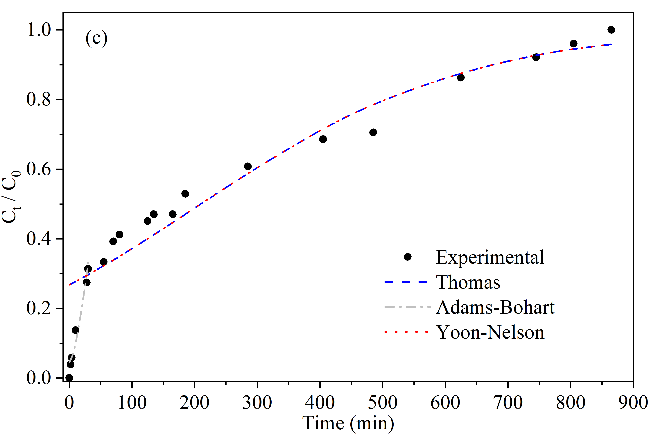 | 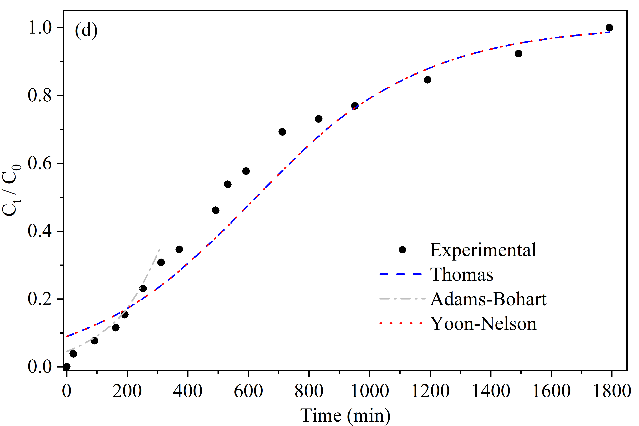 |
| 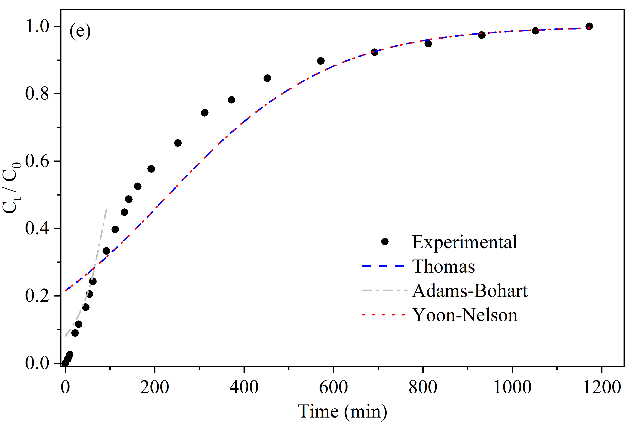 | 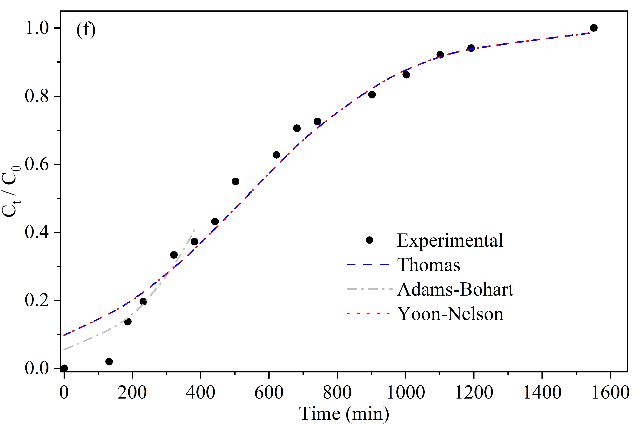 |
| 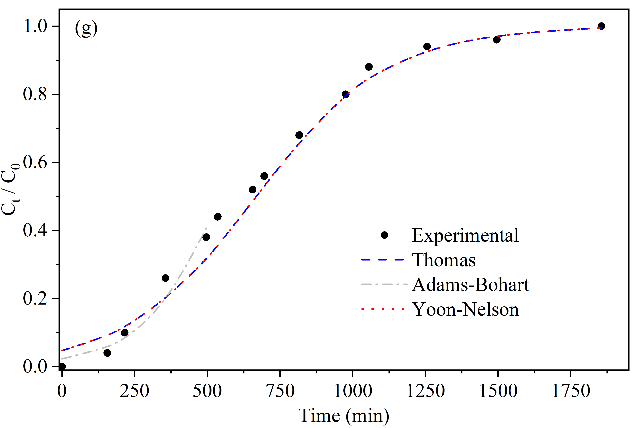 | |
| **Fig. S1. Breakthrough curves modeling by Thomas, Adams-Bohart, and Yoon-Nelson models for the adsorption of P on CWH.** Effect of flow rate and bed height in the breakthrough curves. a: bed height = 2 cm, C_0_ = 50 mg/L, and flow = 0.5 mL/min; b: bed height = 2 cm, C_0_ = 50 mg/L, and flow = 1.0 mL/min; c: bed height = 2 cm, C_0_ = 50 mg/L, and flow = 2.0 mL/min; d: bed height = 2 cm, C_0_ = 25 mg/L, and flow = 2.0 mL/min; e: bed height = 2 cm, C_0_ = 75 mg/L, and flow = 2.0 mL/min; f: bed height = 3 cm, C_0_ = 50 mg/L, and flow = 0.5 mL/min; g: bed height = 4 cm, C_0_ = 50 mg/L, and flow = 0.5 mL/min. | |
